# Effects of bryostatin-1 on chronic myeloid leukaemia-derived haematopoietic progenitors

**DOI:** 10.1038/sj.bjc.6690225

**Published:** 1999-03

**Authors:** S F T Thijsen, G J Schuurhuis, J W van Oostveen, A P Theijsmeijer, K G van der Hem, J H Odding, A M Dräger, G J Ossenkoppele

**Affiliations:** Department of Haematology, BR238, Free University Hospital, De Boelelaan 1117, Amsterdam, 1081 HV Amsterdam, The Netherlands

**Keywords:** CML, bryostatin, purging, CFU-GM, LTCIC, FISH

## Abstract

Bryostatin-1 belongs to the family of macrocyclic lactones isolated from the marine bryozoan *Bugula neritina* and is a potent activator of protein kinase C (PKC). Bryostatin has been demonstrated to possess both in vivo and in vitro anti-leukaemic potential. In samples derived from chronic myeloid leukaemia (CML) patients, it has been demonstrated that bryostatin-1 induces a macrophage differentiation, suppresses colony growth in vitro and promotes cytokine secretion from accessory cells. We investigated the effect of bryostatin-1 treatment on colony-forming unit–granulocyte macrophage (CFU–GM) capacity in the presence of accessory cells, using mononuclear cells, as well as in the absence of accessory cells using purified CD34-positive cells. Cells were obtained from 14 CML patients as well as from nine controls. Moreover, CD34-positive cells derived from CML samples and controls were analysed for stem cell frequency and ability using the long-term culture initiating cell (LTCIC) assay at limiting dilution. Individual colonies derived from both the CFU–GM and LTCIC assays were analysed for the presence of the *bcr–abl* gene with fluorescence in situ hybridization (FISH) to evaluate inhibition of malignant colony growth.

The results show that at the CFU–GM level bryostatin-1 treatment resulted in only a 1.4-fold higher reduction of CML colony growth as compared to the control samples, both in the presence and in the absence of accessory cells. However, at the LTCIC level a sixfold higher reduction of CML growth was observed as compared to the control samples. Analysis of the LTCICs at limiting dilution indicates that this purging effect is caused by a decrease in output per malignant LTCIC combined with an increase in the normal stem cell frequency. It is concluded that bryostatin-1 selectively inhibits CML growth at the LTCIC level and should be explored as a purging modality in CML. © 1999 Cancer Research Campaign


*British Journal of Cancer* (1999) **79**(9/10), 1406–1412
© 1999 Cancer Research Campaign
Article no. bjoc.1998.0225

### Effects of bryostatin-1 on chronic myeloid leukaemia-derived haematopoietic progenitors

**SFT Thijsen, GJ Schuurhuis, JW van Oostveen, AP Theijsmeijer, KG van der Hem, JH Odding, AM Dräger and GJ Ossenkoppele**

Department of Haematology, BR238, Free University Hospital, De Boelelaan 1117, 1081 HV Amsterdam

**Summary** Bryostatin-1 belongs to the family of macrocyclic lactones isolated from the marine bryozoan *Bugula neritina* and is a potent activator of protein kinase C (PKC). Bryostatin has been demonstrated to possess both in vivo and in vitro anti-leukaemic potential. In samples derived from chronic myeloid leukaemia (CML) patients, it has been demonstrated that bryostatin-1 induces a macrophage differentiation, suppresses colony growth in vitro and promotes cytokine secretion from accessory cells. We investigated the effect of bryostatin-1 treatment on colony-forming unit–granulocyte macrophage (CFU–GM) capacity in the presence of accessory cells, using mononuclear cells, as well as in the absence of accessory cells using purified CD34-positive cells. Cells were obtained from 14 CML patients as well as from nine controls. Moreover, CD34-positive cells derived from CML samples and controls were analysed for stem cell frequency and ability using the long-term culture initiating cell (LTCIC) assay at limiting dilution. Individual colonies derived from both the CFU–GM and LTCIC assays were analysed for the presence of the *bcr–abl* gene with fluorescence in situ hybridization (FISH) to evaluate inhibition of malignant colony growth.

The results show that at the CFU–GM level bryostatin-1 treatment resulted in only a 1.4-fold higher reduction of CML colony growth as compared to the control samples, both in the presence and in the absence of accessory cells. However, at the LTCIC level a sixfold higher reduction of CML growth was observed as compared to the control samples. Analysis of the LTCICs at limiting dilution indicates that this purging effect is caused by a decrease in output per malignant LTCIC combined with an increase in the normal stem cell frequency. It is concluded that bryostatin-1 selectively inhibits CML growth at the LTCIC level and should be explored as a purging modality in CML.

**Keywords:** CML; bryostatin; purging; CFU-GM; LTCIC; FISH

Chronic myeloid leukaemia (CML) is a myeloproliferative disease originating in a haematopoietic stem cell and is strongly associated with a cytogenetic abnormality known as the Philadelphia chromosome (Ph) (Nowell and Hungerford, 1960), which is the result of a reciprocal recombination between chromosomes 9 and 22. As a consequence the bcr and the abl proto-oncogenes are fused (Shtivelman et al, 1985). The protein product (p210) of the bcr–abl oncogene has an elevated protein tyrosine kinase (TK) activity and has been implicated in the leukaemogenesis (Gishizky and Witte, 1992). Research trying to modulate CML growth has been focussed mainly on the modulation of the TK function of p210 (Workman, 1992). More specifically, TK inhibitors such as herbimycin A are being investigated (Fukazawa et al, 1991; Pendergast et al, 1991; Druker et al, 1996). However, only a few papers have focussed on the relevance of protein kinase C (PKC) activity in CML. PKC belongs to a large family of serine/threonine kinases (reviewed in Wilkinson and Hallam, 1994). Although p210 itself has no PKC activity the protein can be phosphorylated on serine and threonine residues by PKC enzymes (Pendergast et al, 1987). Furthermore p210 transforming potential is in part dependent on the MAP-kinase pathway which itself is modulated by PKC enzymes (Sawyers et al, 1995).

*Received 23 July 1997*
*Revised 10 September 1998*
*Accepted 25 September 1998*

*Correspondence to:* GJ Ossenkoppele

Bryostatin belongs to the family of macrocyclic lactones isolated from the marine bryozoan *Bugula Neritina* and is a potent activator of PKC (Berkow and Kraft, 1985; Pettit et al, 1986; Wender et al, 1988; Kraft, 1993). After binding of bryostatin, PKC translocates to the plasma membrane and starts phosphorylating target proteins on serine and threonine residues (Wilkinson and Hallam, 1994) thereby altering protein activity and influencing signal transduction events (reviewed in Dekker and Parker, 1994). Bryostatin has been demonstrated to possess both in vivo and in vitro anti-leukaemic potential (Jones et al, 1990; Grant et al, 1991; reviewed in Steube and Drexler, 1993). Our group has shown that in acute myeloid leukaemia bryostatin can reduce leukaemic outgrowth depending on concentration and time of exposure whereas it can stimulate normal outgrowth (van der Hem et al, 1995, 1996). Differential effects found on normal and malignant haematopoiesis (van der Hem et al, 1995) might be explained by bryostatin-induced secretion of cytokines in accessory cells such as monocytes (Lilly et al, 1990; Steube and Drexler, 1995; van der Hem et al, submitted for publication) and stromal cells in long-term bone marrow cultures (Lilly et al, 1996). The release of GM–CSF, IL-3 and other related cytokines have been suggested to stimulate non-leukaemic growth whereas the induction of TNF-$\alpha$ might suppress leukaemic growth (Steube and Drexler, 1995).

In samples derived from CML patients it has been demonstrated that bryostatin-1 induces macrophage differentiation and suppresses colony growth in vitro (Lilly et al, 1990). Bryostatin-1 has also been shown to induce cell cycle arrest correlating with dephosphorylation of CDK2 and upregulation of P21 as demonstrated with the U937 cell line and CML-derived cells (Asiedu et al, 1995).

In view of these findings we investigated the effect of bryostatin-1 treatment on CFU–GM capacity in the presence of accessory cells using unseparated mononuclear cells and in the absence of accessory cells using purified CD34-positive cells. Cells were obtained from 14 CML patients as well as from nine controls. Moreover, CD34-positive cells derived from CML samples and controls were analysed for stem cell frequency and ability using the LTCIC assay at limiting dilution. Individual colonies derived from these assays were analysed by fluorescent in situ hybridization (FISH) for the presence of the *bcr–abl* gene to evaluate selective inhibition of malignant colony growth.

### MATERIALS AND METHODS

### Patients and cell processing

Bone marrow was obtained from CML patients in first chronic phase: unique patient number (UPN) 803, 906, 976, 1065, 1121, 1144, 1162, and from CML patients in blastic phase (BP): UPN 633 and 765. Samples UPN 971 and 1000 were obtained via leukapheresis from CML patients in chronic phase. Samples UPN 932, 1054, 1055, 1061, 1062, 1067, 1071, 1151 and 1152 were obtained from chronic phase CML patients via leukapheresis after induction chemotherapy and granulocyte colony stimulating factor (G-CSF, Filgrastim, Amgen, Breda, The Netherlands) administration. Some of the total of 14 CML patients were sampled more than once and each sample received its own UPN number. Control bone marrows were obtained from seven healthy volunteers and two control leukapheresis samples were obtained from patients with breast cancer after peripheral stem cell mobilization. All samples were obtained after informed consent. Mononuclear cells were isolated from the samples by Ficoll-Paque (Pharmacia, Upsala, Sweden) density gradient centrifugation ($\delta = 1.077\ \boldsymbol{g}\ cm^{-3}$). Remaining red cells were lysed with ammonium chloride. CD34-positive cells were isolated using the MiniMacs system (Miltenyi Biotec, Bergisch Gladbach, Germany) according to the instructions of the manufacturer. After two consecutive passages over two separation columns, purity was always more than 90% as determined by FACS analysis.

### Bryostatin-1 incubation

Bryostatin-1 was generously provided by Dr GR Petitt (Arizona State University, Tempe, AZ, USA). Stock solutions of $10^{-3}$ and $10^{-4}$ M were made in dimethyl sulphoxide (DMSO) and stored at –30°C until use. The cells were incubated at the desired bryostatin-1 concentrations in Roswell Park Memorial Institute (RPMI), 10% fetal calf serum (FCS) (Gibco, Grand Island, NY, USA) for the desired pre-incubation period. After culturing the cells were counted and scored with trypan blue exclusion for viability. The final concentration of DMSO in cell culture media never exceeded 0.25%, a concentration which was determined to have no effect on the CFU–GM capacity (data not shown).

### Progenitor assays

#### *CFU–GM*

CD34-positive cells were cultured in semi-solid medium in the presence of 5% human placenta conditioned medium (HPCM) (Burgess et al, 1977) and 10% FCS (Gibco). Cultures were incubated at 37°C in 5% carbon dioxide ($CO_2$). The cells were plated at four different concentrations: 500, 1000, 5000 and 10 000 CD34-positive cells per ml CFU–GM medium, in triplicate to optimize colony density for quantification and colony harvesting. Colonies (> 40 cells) and clusters (8–40 cells) were counted at day 12 and were plucked at random from different cultures with a vacuum capillary system. The colony size was about 500 cells.

#### *LTCIC (Sutherland* et al*, 1990)*

Human bone marrow cultures were initiated with samples obtained from healthy volunteers in long term bone marrow culture (LTBMC) medium (Coulombel et al, 1983): alfa medium supplemented with 12.5% horse serum, 12.5% FCS, $10^{-4}$ M β-mercaptoethanol, $10^{-6}$ M hydrocortisone sodium succinate, glutamine, penicillin and streptomycin (all supplements from Gibco). After 2 weeks of culturing the adherent cells were harvested using trypsin (Gibco), irradiated with 13 Gy ($^{60}Co$) and plated in 96-well plates (Costar, Cambridge, MA, USA) at $1.25 \times 10^4$ cells in 200 µl LTBMC medium. After 2–7 days CD34-positive cells were plated on top of the stromal cell layer and underwent a weekly half-medium change. Cultures were incubated at 37°C in 5% $CO_2$. After 5 weeks the non-adherent and adherent cells (using trypsin) were harvested and the CFU–GM capacity of cells was determined as described earlier. The linearity between input of CD34-positive cells and CFU–GM output of LTCICs was determined: long-term cultures initiated with different concentrations of CD34-positive cells (ranging from 1000 to 20 000 per well) were harvested and plated in the CFU–GM assay, each at three different concentrations. Experiments were done in triplicate. In addition for some samples the frequency of the LTCIC cells (number of LTCICs per CD34-positive cell) was determined with the limiting dilution assay. CD34-positive cells were plated at concentrations of 50, 150, 500 and 1000 cells/well. After culturing for 5 weeks the cells were harvested using trypsin and were subsequently plated in the CFU–GM assay and scored after 2 weeks for positive/negative results. The LTCIC frequencies were determined with Poisson statistics (Sutherland et al, 1990). Combination of LTCIC-derived CFU–GM output per CD34-positive cell with the frequency of LTCIC per CD34-positive cell leads to the CFU–GM output/single LTCIC: (CFU–GM output/CD34-positive cell)/(LTCIC/CD34-positive cell) = (CFU–GM output/single LTCIC).

### Molecular analysis

Interphase-FISH (I-FISH) was performed as described earlier (Thijsen et al, 1997). Briefly, cells derived from a single colony were put on a glass slide, fixed in 70% ethanol and dipped in a 0.1% gelatine (Sigma, St Louis, MO, USA) solution (Lakhotia et al, 1993) incubated with 0.1 mg $ml^{-1}$ RNAse A (Boehringer Mannheim, Mannheim, Germany) followed by 0.01% pepsin (Sigma). Post-fixation the slides were dehydrated and denatured at 72°C in 70% formamide (Baker, Deventer, The Netherlands). Cosmids bcr 51 and abl 18 (a kind gift from Dr G Grosveld and Dr A Hagemeijer, Erasmus University, Rotterdam, The Netherlands) were labelled by nick-translation with digoxigenin11-dUTP (Boehringer Mannheim) and biotin-14-dATP (Gibco), respectively (Arnoldus et al, 1990). An amount of 15 ng of each probe was mixed with a 100-fold excess of Hu-cot-1 DNA (Gibco) in 50% formamide, 2 × SSC and 10% dextran sulphate (Sigma). This solution was denatured at 74°C for 4 min, cooled on ice and incubated at 37°C for 2 h and subsequently layered

*© Cancer Research Campaign 1999*

onto the slides and sealed with rubber cement. After an overnight hybridization at 37°C the cells were washed at 40°C with 55% formamide in 2 × SSC, followed by 0.1 × SSC at 60°C. The biotin labelled probe was detected with fluorescein avidin DN followed by biotinylated anti-avidin and fluorescein avidin DN (all obtained from Vector Laboratories Inc., Burlingame, CA, USA). The digoxigenin labelled probe was detected with anti-digoxigenin–rhodamine (Boehringer Mannheim) followed by Texas red-conjugated donkey anti-sheep (Jacksons Immuno-research, West Grove, PA, USA). After dehydration slides were embedded using Vectashield (Vector Laboratories Inc.) containing 0.1 μg ml$^{-1}$ dapi (Sigma). Samples were scored with an Axioskop 50 (Carl Zeiss Jena GmBH, Jena, Germany) using a triple band pass filter (Omega Optical, Brattleboro, VT, USA). Nuclei were scored positive for the *bcr–abl* gene when a green and a red spot were less than one spot diameter apart. A colony was scored when at least ten nuclei could be evaluated. More than 90% of the evaluable nuclei of a single colony should generate the same (positive or negative) result, otherwise a colony was considered not evaluable. On average 100 nuclei per colony were evaluable.

### RESULTS

### Dose finding of bryostatin-1

The effect of bryostatin-1 treatment on the CFU–GM capacity of cells from three CML patient bone marrow samples and three control bone marrow samples was measured with the CFU–GM assay. At time of sampling these patients were in advanced chronic phase or in blastic phase as to make sure that the entire cell population would be Ph-positive. The cells were pre-incubated for 24, 48, 72 and 96 h with 0, 10, 100, 250 and 500 nM bryostatin-1. The readouts of the CFU–GM assays indicate a higher sensitivity of CML-derived samples as compared to their normal counterparts. These effects hold true especially for 250 and 500 nM and are already achieved after 24 h of pre-incubation. More than a tenfold increase in sensitivity of CML progenitors was observed towards treatment with bryostatin as compared to the normal cells (data not shown). This led us to analyse more samples in detail for the effects of 24-hour pre-incubation with 250 nM bryostatin-1 on normal and malignant haematopoiesis.

### Effects of pre-incubation with 250 nM bryostatin-1 on CFU–GM and LTCIC capacity

A total of 1000 CML-derived mononuclear cells generated a mean output of 16 colonies/clusters in the CFU–GM assay ($n$ = 9). For the control samples this number was 10 ($n$ = 6). A total of 100 CML-derived CD34-positive cells generated a mean output of 23 colonies/clusters in the CFU–GM assay ($n$ = 8). For the CD34-positive cells derived from the control samples this number was 20 ($n$ = 5). The readouts of the CFU–GM assays show that upon treatment with 250 nM bryostatin-1, in the presence of accessory cells, on average 39% of the CFU–GM capacity remained as compared to the CML cells pre-incubated without bryostatin-1. It should be mentioned that four of these CML samples were analysed fresh and five were analysed after cryopreservation and thawing. Both groups showed a very similar bryostatin-1-induced reduction of CFU–GM capacity. For the control samples, 56% of the clonogenic capacity remained (Figure 1). The clonogenic capacity of

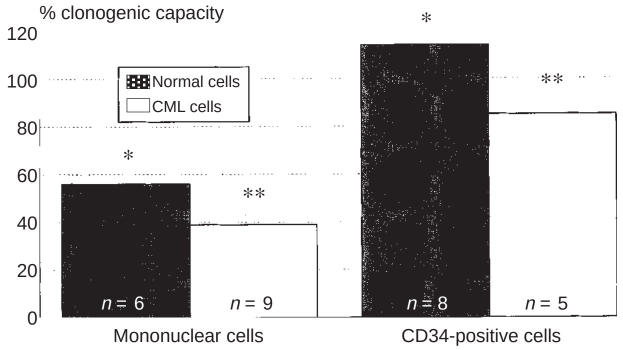

**Figure 1** Effects of bryostatin pre-incubation on CFU–GM capacity. *,** Statistically different from corresponding symbol; < 0.05 student's *t*-test

CD34-positive cells after treatment with bryostatin-1 was reduced to 85% for the CML-derived samples, as compared to the CML cells pre-incubated without bryostatin-1. For the control samples a mean increase to 114% in clonogenic capacity could be observed (Figure 1). Neither the unseparated mononuclear cells nor the purified CD34-positive cells showed significant differences between the CML samples and control samples. However, the bryostatin-1-induced reduction of CFU–GM capacity was significantly higher ($P < 0.05$, Student's $t$-test) in the presence of accessory cells, both for the CML and control samples.

At the LTCIC level 100 CML-derived CD34-positive cells generated a mean output of 43 colonies/clusters ($n$ = 6). For the CD34-positive cells derived from the control samples, this number was 38 ($n$ = 5) (Table 2). The readouts of the LTCIC assays indicate a highly significant difference in effects of bryostatin-1-induced growth modulation on normal and malignant haematopoiesis ($P < 0.01$, Student's $t$-test). CD34-positive cells derived from control samples were not affected significantly by bryostatin-1 treatment, whereas CD34-positive cells derived from CML samples showed a sixfold reduction in LTCIC-derived CFU–GM output.

### Molecular analysis of individual CFU–GM and LTCIC colonies

In total, 799 colonies were analysed with FISH for the presence of the *bcr–abl* gene. In a previous study, we analysed 160 colonies pairwise both with reverse transcriptase polymerase chain reaction (RT–PCR) (for the presence of the bcr–abl mRNA) and FISH (for the presence of the *bcr–abl* gene) (Thijsen et al, 1997). We demonstrated a highly significant correlation between both techniques.

At the CFU–GM level the molecular analysis of individual CFU–GM colonies, generated by the unseparated mononuclear cells, showed a mixture of bcr–abl-positive and -negative colonies in three out of eight cases (Table 1). Upon bryostatin-1 treatment, no significant change in the ratio between bcr–abl-positive/negative colonies was observed in cases UPN 1055 and 1152. However, a statistically significant ($P < 0.05$, $\chi^2$ test) relative loss of bcr–abl-positive colonies in case UPN 1151 was detected while in sample UPN 1162, a bcr–abl-negative colony could only be detected after bryostatin-1 treatment. Concerning the CD34-positive cells, no shift was observed in case UPN 1055. A significant increase in the number of bcr–abl-negative colonies could be observed in case UPN 906 ($P < 0.05$, $\chi^2$ test) while for UPN 971 a bcr–abl-negative colony could only be detected after bryostatin-1

 © Cancer Research Campaign 1999

**Table 1** Effects of bryostatin-1: molecular analysis of individual CFU–GM colonies[a]

| Mononuclear cells | | | | | CD34-positive cells | | | | |
|---|---|---|---|---|---|---|---|---|---|
| | bcr–abl +/– colonies[a] | | | | | bcr–abl +/– colonies[a] | | | |
| **UPN** | **– bryo** | | **+ bryo** | | **UPN** | **– bryo** | | **+ bryo** | |
| 932[c] | 17/0 | | 16/0 | | 932 | 24/0 | | 50/0 | |
| 1054 | 5/0 | | 8/0 | | 1054 | 6/0 | | 9/0 | |
| 1055[c] | 13/13 | (50)[b] | 10/21 | (68) | 1055 | 4/5 | (56) | 5/5 | (50) |
| 1121 | 8/0 | | 23/0 | | 1121 | 23/0 | | 20/0 | |
| 1144 | 12/0 | | 2/0 | | 1144 | 16/0 | | 14/0 | |
| 1151[c] | 10/10 | (50) | 2/20 | (91)[d] | 996 | 29/0 | | 1/0 | |
| 1152[c] | 1/24 | (96) | 1/12 | (92) | 1000 | 23/0 | | 27/0 | |
| 1162[c] | 20/0 | (0) | 17/1 | (6) | 971 | 18/0 | (0) | 35/1 | (3) |
| | | | | | 906 | 22/6 | (21) | (19/20) | (51)[d] |
| No col | 96/47[e] | | 63/54 | | | 163/11 | | 180/26 | |

[a]No. of bcr–abl (positive = +; negative = –) CFU–GM colonies. [b]No. in parentheses indicate the percentages [c]Analysed after cryopreservation and thawing. [d]Significantly different from the control ($P < 0.01$, $\chi^2$ test). [e]No. of colonies (in total 640).

**Table 2** Effects of bryostatin-1: clonogenic capacity and molecular analysis of individual LTCIC-derived colonies[a]

| | LTCIC-derived CFU–GM output/100 CD34 + cells[a] | | | bcr–abl +/– colonies[b] | |
|---|---|---|---|---|---|
| **UPN** | **– bryo** | **+ bryo** | | **– bryo** | **+ bryo** |
| 932 | 3.6 | 0.6 | (17)[d] | 5/0 | 8/0 |
| 1000 | 3.0 | 1.0 | (33) | 3/0 | 6/0 |
| 1054 | 98.0 | 12.7 | (13) | 18/0 | 16/0 |
| 1121 | 38.0 | 8.4 | (22) | 13/0 (0) | 13/7 (35)[e] |
| 1144 | 51.6 | 2.2 | (4) | 17/0 | 18/0 |
| 932[c] | 63.6 | 8.2 | (13) | 19/0 | 16/0 |
| | | | | 75/0[i] | 77/7 |
| mean ± SEM | 43 ± 15 | 5.5 ± 2.0 | (17 ± 4)[f] | | |
| CM4[g] | 1.3 | 2.3 | (177) | | |
| CM5 | 0.6 | 0.17 | (28) | | |
| CM6 | 56 | 54 | (96) | | |
| LF1[h] | 89 | 84 | (94) | | |
| LF2 | 43.9 | 51.1 | (116) | | |
| mean ± SEM | 38 ± 17 | 38 ± 16 | (102 ± 24) | | |

[a]No. of LTCIC-derived CFU–GM output/100 CD34-positive cells. [b]No. of bcr–abl (Positive = +; negative = –) LTCIC-derived CFU–GM colonies. [c]Analysed after cryopreservation and thawing. [d]No. in parentheses indicate the percentages. [e]Statistically different from the untreated control ($P < 0.05$, $\chi^2$ test). [f]Statistically different from the control samples ($P < 0.01$, Students *t*-test). [g]CM, bone marrow samples obtained from three healthy volunteers. [h]LF, leukapheresis samples obtained from patients with non-hematologic malignancy after stem cell mobilization. [i]No. of colonies (in total 159).

treatment. At the LTCIC level (Table 2) six samples were analysed. Bcr–abl-negative colonies were detected in a sample derived from UPN 1121 after bryostatin-1 treatment whereas without bryostatin treatment, no bcr–abl-negative colonies were detected.

### Effects of bryostatin-1 pre-incubation on LTCIC frequency and CFU–GM output/LTCIC

To determine the effect of bryostatin-1 treatment on the stem cell frequency and CFU–GM output/LTCIC, CD34-positive cells from four CML samples and three control samples were analysed with the LTCIC assay at limiting dilution. The four CML samples showed a statistically significant ($P < 0.05$ Student's *t*-test) 7.7-fold reduction in LTCIC-derived CFU–GM output per CD34-positive cell (down to 13%) and an average stem cell frequency reduction to 70% and was detected (Table 3); this indicates that bryostatin-1 reduces the CFU–GM output per LTCIC 5.3-fold (down to 19%). The three control samples showed no reduction in the LTCIC-derived CFU–GM output per CD34-positive cell and since an increase in the stem cell frequency was observed (up to 236%) this indicates only a moderate decrease to 66% in the CFU–GM output per LTCIC.

The bryostatin-1 experiments have been performed after pre-incubation for 24 h with or without bryostatin-1. However, when comparing the CFU–GM output per LTCIC in the CML samples pre-incubated without bryostatin-1 with fresh samples, a mean

*© Cancer Research Campaign 1999*

**Table 3** The effect of bryostatin-1 and freezing on LTCIC frequency, LTCIC-derived CFU–GM output/CD34-positive cell and CFU–GM output/LTCIC[b]

| | LTCIC-derived CFU-GM/CD34 + cell[a] | | LTCIC/CD34+ cell[b] | | CFU-GM/LTCIC[c] | |
|---|---|---|---|---|---|---|
| | – bryostatin[f] | + bryostatin[f] | – bryostatin | + bryostatin | – bryostatin | + bryostatin |
| CML ($n = 4$) | 0.63 ± 0.13 | 0.13 ± 0.02[e] (13)[d] | 1/416 ± 0.0008 | 1/607 ± 0.001 (70) | 312 ± 60 | 55 ± 18[e] (19) |
| Controls ($n = 3$) | 0.63 ± 0.13 | 0.63 ± 0.11 (102) | 1/179 ± 0.002 | 1/70 ± 0.009 (236) | 122 ± 22 | 90 ± 38 (66) |
| | Fresh | Frozen | Fresh | Frozen | Fresh | Frozen |
| Unpaired ($n = 6$) | 0.25 ± 0.1 | 0.63 ± 0.13 | 1/194 ± 0.002 | 1/416 ± 0.008 | 50 ± 9 | 312 ± 60[e] |
| Paired ($n = 3$) | 0.77 ± 0.23 | 0.68 ± 0.16 (99) | 1/133 ± 0.0008 | 1/293 ± 0.0006 (47) | 98 ± 24 | 210 ± 60 (212) |

[a]LTCIC frequency expressed as the number of LTCIC per CD34-positive cell. [b]No. of LTCIC-derived CFU–GM colonies/clusters generated per CD34-positive cell. [c]No. of CFU–GM colonies/clusters generated by 1 LTCIC. [d]No. in parentheses indicate the percentages. [e]Significantly different from the control ($P < 0.05$, Student's *t*-test). [f]Positive = +; negative = –.

sixfold higher output (312 vs 50 colonies and clusters) was observed (Table 3), accompanied by a decrease of stem cell frequency (1/416 vs 1/194). Since the only difference was the culturing period of 24 h, three samples were analysed pairwise. In each case a 24-hour pre-incubation led to a twofold increase in the CFU–GM output per LTCIC and a decrease in the stem cell frequency.

### DISCUSSION

The clonogenic capacity at the CFU–GM level showed an average 1.4-fold higher reduction after bryostatin-1 treatment in the CML samples as compared to the control samples, both for the CD34-positive cells and the unseparated mononuclear cells. These differences were not significant. Molecular analysis of individual colonies showed a significant elimination of bcr–abl-positive colonies in only two out of seven samples containing bcr–abl-negative colonies and therefore supported the idea that the observation does reflect a true biological difference. These findings are in line with the findings of Lilly et al (1990), who also demonstrated a reduction in CML colony growth after pre-incubation with bryostatin. Using CD34-positive cells, our results show an only limited stimulation of CFU–GM outgrowth of control cells after bryostatin pre-incubation, although in one bone marrow sample we observed an almost twofold stimulation. These findings are in contrast to others who observed a strong stimulation of normal CFU–GM (Jones et al, 1990; Grant et al, 1991).

Remarkably, a significant ($P < 0.05$ Student's *t*-test) twofold higher reduction upon bryostatin-1 treatment was demonstrated in samples using mononuclear cells as compared to the experiments using CD34-positive cells, both with CML and control samples. Unfortunately, the reduction at the CFU–GM level is the same for CML and control samples and thus the presence of accessory cells does not lead to selective loss of malignant cells. Since bryostatin-1 induced release of TNF-α, from accessory cells such as monocytes (Steube and Drexler, 1995; van der Hem et al) might play a role in this phenomenon it can be argued that bcr–abl-positive CFU–GMs are equally sensitive to TNF-α as control cells. A similar effect has been described by the current authors for resistance to TNF-α-mediated apoptosis induction in the bcr–abl-positive K562 cell line (Legdeur et al, 1996).

At the LTCIC level, we show evidence of a differential effect of bryostatin-1 treatment on malignant vs normal haematopoiesis. CML samples showed a sixfold reduction in LTCIC-derived CFU–GM output per CD34-positive cell whereas bryostatin-1 treatment did not affect the control samples. These results are highly significant. Molecular analysis of individual colonies was unrewarding since only one sample contained a mixture of bcr–abl-positive and -negative colonies. Interestingly these bcr–abl-negative colonies were detected after bryostatin-1 treatment. These results underline the importance of including assays for uncommitted progenitors since purging effects of bryostatin-1 would have been largely obscured when cells were analysed with the CFU–GM assay only.

LTCIC experiments were performed with CD34-positive cells and, thus, bryostatin-1 may have a direct effect on the LTCICs, for example by modulating the MAP kinase pathway (Sawyers et al, 1995). In this respect, the higher sensitivity of CML-derived LTCICs as compared to the CML-derived CFU–GMs might be explained by the possible absence of p210 in Ph-positive stem cells (Bedi et al, 1993), leading to a possible increased vulnerability for apoptosis of LTCICs compared to CFU–GMs (Bedi et al, 1994; McGahon et al, 1994). To study the effects of bryostatin-1

 *© Cancer Research Campaign 1999*

treatment in more detail, seven samples were analysed with the LTCIC assay at limiting dilution. Analysis of four CML samples showed that the observed effects are caused by a reduction in CFU–GM output per LTCIC, while the reduction in stem cell frequency is much less pronounced. In the three controls an overall unchanged LTCIC-derived CFU–GM output per CD34-positive cell was observed. The analysis at limiting dilution showed a twofold increase in LTCIC frequency and a moderately reduced CFU–GM output per LTCIC. Taken together, purging would thus be a combined effect of a strongly reduced CFU–GM output per malignant LTCIC and an increased normal stem cell frequency. In future bryostatin could be explored as a possible purging agent in the setting of autologous stem cell transplantation for those CML patients not eligible for allogenic stem cell transplantation. Moreover, dose finding studies for bryostatin have been performed opening the way for in vivo purging strategies (Jayson et al, 1995; Varterasian et al, 1998; Grant et al, 1998).

Remarkably, pre-incubation without bryostatin-1 resulted in a higher CFU–GM output per LTCIC and a lower LTCIC frequency as compared to samples not pre-incubated at all. This indicates that culturing of cells on stromal feeder layer is very different from culturing in suspension. Further studies should be performed to investigate whether these observations might play a role in the purging effect of bone marrow cultures (Barnett et al, 1994). In conclusion, bryostatin-1 selectively inhibits CML growth, especially at the LTCIC level, and bryostatin-1 modulation of haematopoiesis should be explored as a purging modality in CML.

### REFERENCES

Arnoldus EP, Wiegant J, Noordermeer IA, Wessels JW, Beverstock GC, Grosveld GC, van der Ploeg M and Raap AK (1990) Detection of the Philadelphia chromosome in interphase nuclei. *Cytogen Cell Genet* **54**: 108–111

Asiedu C, Biggs J, Lilly M and Kraft AS (1995) Inhibition of leukemic cell growth by the protein kinase C activator bryostatin 1 correlates with the dephosphorylation of cyclin-dependent kinase 2. *Cancer Res* **55**: 3716–3720

Barnett MJ, Eaves CJ, Phillips GL, Gascoyne RD, Hogge DE, Horsman DE, Humphries RK, Klingemann HG, Lansdorp PM, Nantel SH, Reece DE, Shepherd JD, Spinelli JJ, Sutherland HJ and Eaves AC (1994) Autografting with cultured marrow in chronic myeloid leukemia: results of a pilot study. *Blood* **84**: 724–732

Bedi A, Zehnbauer BA, Collector MI, Barber JP, Zicha MS, Sharkis SJ and Jones RJ (1993) BCR–ABL gene rearrangement and expression of primitive hematopoietic progenitors in chronic myeloid leukemia. *Blood* **81**: 2898–2902

Bedi A, Zehnbauer BA, Barber JP, Sharkis SJ and Jones RJ (1994) Inhibition of apoptosis by BCR–ABL in chronic myeloid leukemia. *Blood* **83**: 2038–2044

Berkow RL and Kraft AS (1985) Bryostatin, a non-phorbol macrocyclic lactone, activates intact human polymorphonuclear leukocytes and binds to the phorbol ester receptor. *Biochem Biophys Res Commun* **131**: 1109–1116

Burgess AW, Wilson EMA and Metcalf D (1977) Stimulation by human placental conditioned medium of hemopoietic colony formation by human marrow cells. *Blood* **49**: 573–583

Coulombel L, Kalousek DK, Eaves CJ, Gupta CM and Eaves AC (1983) Long-term marrow culture reveals chromosomally normal hematopoietic progenitor cells in patients with Philadelphia chromosome-positive chronic myelogenous leukemia. *N Engl J Med* **308**: 1493–1498

Dekker LV and Parker PJ (1994) Protein kinase C–a question of specificity. *Trends Biochem Sci* **19**: 73–77

Dräger AM, van der Hem KG, Huijgens PC, Odding JH and Schuurhuis GJ (1999) Role of TNF alfa in bryostatin-induced inhibition of human hematopoiesis. *Leukemia*, in press

Druker BJ, Tamura S, Buchdunger E, Ohno S, Segal GM, Fanning S, Zimmermann J and Lydon NB (1996) Effects of a selective inhibitor of the abl tyrosine kinase on the growth of bcr–abl-positive cells. *Nat Med* **2**: 561–566

Fukazawa H, Li PM, Yamamoto C, Murakami Y, Mizuno S and Uehara Y (1991) Specific inhibition of cytoplasmic protein tyrosine kinases by herbimycin A in vitro. *Biochem Pharm* **42**: 1661–1671

Gishizky ML and Witte ON (1992) Initiation of deregulated growth of multipotent progenitor cells by bcr-abl in vitro. *Science* **256**: 836–839

Grant S, Pettit GR, Howe C and McCrady C (1991) Effect of the protein kinase C activating agent bryostatin 1 on the clonogenic response of leukemic blast progenitors to recombinant granulocyte-macrophage colony-stimulating factor. *Leukemia* **5**: 392–398

Grant S, Roberts J, Poplin E, Tombes MB, Kyle B, Welch D, Carr M and Bear HD (1998) Phase Ib trial of bryostatin 1 in patients with refractory malignancies *Clin Cancer Res* **4**: 611–618

Jayson GC, Crowther D, Prendiville J, McGown AT, Scheid C, Stern P, Young R, Brenchley P, Chang J, Owens S and Pettit GR (1995) A phase I trial of bryostatin 1 in patients with advanced malignancy using a 24 hour intravenous infusion. *Br J Cancer* **72**: 461–468

Jones RJ, Sharkis SJ, Miller CB, Rowinsky EK, Burke PJ and May WS (1990) Bryostatin 1, a unique biologic response modifier: anti-leukemic activity in vitro. *Blood* **75**: 1319–1323

Kraft AS (1993) Bryostatin 1: will the oceans provide a cancer cure? *J Natl Cancer Inst* **85**: 1790–1792

Lakhotia SC, Sharma A, Mutsuddi M and Tapadia MG (1993) Gelatin as a blocking agent in Southern blot and chromosomal in situ hybridizations. *Trends Genet* **9**: 261

Legdeur MC, Bontje PM, Ossenkoppele GJ, Beelen RH, van de Loosdrecht AA, Broekhoven MG, Langenhuijsen MM, Thijsen SF, Hofstee H and Schuurhuis GJ (1996) The role of BCL-2 and bax protein in monocyte-mediated apoptosis in human leukemic cell lines. *Exp Hematol* **24**: 1530–1539

Lilly M, Tompkins C, Brown C, Pettit G and Kraft A (1990) Differentiation and growth modulation of chronic myelogenous leukemia cells by Bryostatin. *Cancer Res* **50**: 5520–5525

Lilly M, Vo K, Le T and Takahashi G (1996) Bryostatin 1 acts synergistically with interleukin-1-alpha to induce secretion of G-CSF and other cytokines from marrow stromal cells. *Exp Hematol* **24**: 613–621

McGahon A, Bissonnette R, Schmitt M, Cotter KM, Green DR and Cotter TG (1994) BCR–ABL maintains resistance of chronic myelogenous leukemia cells to apoptotic cell death. *Blood* **83**: 1179–1187

Nowell PC and Hungerford DA (1960) A minute chromosome in human chronic granulocytic leukemia. *Science* **132**: 1497

Pendergast AM, Traugh JA and Witte ON (1987) Normal cellular and transformation-associated abl proteins share common sites for protein kinase C phosphorylation. *Mol Cell Biol* **7**: 4280–4289

Pendergast AM, Muller AJ, Havlik MH, Clark R, McCormick F and Witte ON (1991) Evidence for regulation of the human ABL tyrosine kinase by a cellular inhibitor. *Proc Natl Acad Sci USA* **88**: 5927–5931

Pettit GR, Kamano Y and Herald CL (1986) Antineoplastic agents 118. Isolation and structure of bryostatin 9. *J Nat Prod* **49**: 661–664

Sawyers CL, McLaughlin J and Witte ON (1995) Genetic requirement for ras in the transformation of fibroblasts and hematopoietic cells by the bcr-abl oncogene. *J Exp Med* **181**: 307–313

Shtivelman E, Lifshitz B, Gale RP and Canaani E (1985) Fused transcripts of abl and bcr genes in chronic myelogenous leukaemia. *Nature* **315**: 550–554

Steube KG and Drexler HG (1993) Differentiation and growth modulation of myeloid leukemia cells by the protein kinase C activating agent bryostatin-1. *Leuk Lymphoma* **9**: 141–148

Steube KG and Drexler HG (1995) The protein kinase c activator bryostatin-1 induces the rapid release of TNF-alpha from mono-mac-6 cells. *Biochem Biophys Res Commun* **214**: 1197–1203

Sutherland HJ, Lansdorp PM, Henkelman DH, Eaves AC and Eaves CJ (1990) Functional characterization of individual human hematopoietic stem cells cultured at limiting dilution on supportive marrow stromal layers. *Proc Natl Acad Sci USA* **87**: 3584–3588

Thijsen SFT, Schuurhuis GJ, van Oostveen JW, Theijsmeijer AP, Langenhuijsen MMAC and Ossenkoppele GJ (1997) Molecular analysis of hematopoietic colonies derived from chronic myeloid leukemia patients: interphase fluorescence in situ hybridisation compared with RT-PCR. *Leukemia* **11**: 301–305

van der Hem KG, Drager AM, Odding JH, Langenhuijsen MM and Huijgens PC (1995) Bryostatin-5 stimulates normal human hematopoiesis and inhibits proliferation of HL60 leukemic cells. *Leuk Res* **19**: 7–13

van der Hem KG, Schuurhuis GJ, Drager AM, Odding JH and Huijgens PC (1996) Heterogenous effects of bryostatin on human myeloid leukemia clonogenicity: dose and time scheduling dependency. *Leuk Res* **20**: 743–750

© Cancer Research Campaign 1999 

Varterasian ML, Mohammad RM, Eilender DS, Hulburd K, Rodriguez DH, Pemberton PA, Pluda JM, Dan MD, Pettit GR, Chen BD and Al-Katib AM (1998) Phase I study of bryostatin 1 in patients with relapsed non-Hodgkin's lymphoma and chronic lymphocytic leukemia. *J Clin Oncol* **16**: 56–62

Wender PA, Cribbs CM, Koehler KF, Sharkey NA, Herald CL, Kamano Y, Pettit GR and Blumberg PM (1988) Modeling of the bryostatins to the phorbol ester pharmacophore on protein kinase C. *Proc Natl Acad Sci USA* **85**: 7197–7201

Wilkinson SE and Hallam TJ (1994) Protein kinase C: is its pivotal role in cellular activation over-stated? *Trends Pharmacol Sci* **15**: 53–57

Workman P (1992) Signal transduction inhibitors as novel anticancer drugs: where are we? *Ann Oncol* **3**: 527–531

 *© Cancer Research Campaign 1999*
